# An Unprecedented Experience: Personal and Socio-Political Impacts of the COVID-19 Lockdown in Saudi Arabia

**DOI:** 10.7759/cureus.54857

**Published:** 2024-02-25

**Authors:** Ghadah Alkhaldi

**Affiliations:** 1 College of Applied Medical Sciences/Community Health Sciences, King Saud University, Riyadh, SAU

**Keywords:** early stage of pandemic, qualitative studies, cultural context, people experience, covid 19 impact of lockdown

## Abstract

The COVID-19 pandemic provided an opportunity to understand the experiences of different cultures and the application of preventive measures during a pandemic. That understanding can lead to the development of important evidence to grasp the global situation and prepare for future health crises. This qualitative study explores the impact of COVID-19 lockdown measures on individuals in Saudi Arabia, focusing on personal and socio-political effects. Employing semi-structured interviews with 28 participants, the research delves into the lived experiences during the early stages of the pandemic, highlighting emotional coping mechanisms, behavioral changes, role responsibility adaptations, and perceptions of governmental actions. The findings reveal a spectrum of emotional responses, from worry and fear to acceptance and contentment, and significant shifts in behavior and societal norms. The study underscores the importance of understanding cultural contexts in pandemic responses and offers insights into the resilience and adaptability of the Saudi community. It suggests the need for tailored interventions that consider the complex interplay of emotional, behavioral, and socio-political factors in managing future health crises.

## Introduction

The COVID-19 pandemic created major disruptions around the world, and it was the harbinger of a number of experiences that the world was not properly prepared for, the lockdown being the main one of those. Countries implemented lockdowns differently, whether partial or full lockdown and the period between them [1]. In addition, for each country, the impact on mental health, the spread of COVID-19, the economy, lifestyle, education, and environmental health were different, alongside the perceptions of the populations [2-9].

Saudi Arabia started to apply preventive measures even before the first announced case in the country, applying strict measures including but not limited to closing international borders, quarantine for arrivals, converting to education online, closing all recreational facilities, and applying partial or complete lockdown to different parts of the kingdom depending on disease status [10].

Research on Saudi Arabia's experience at the beginning of the pandemic focused on the use of reviews, clinical studies, and/or cross-sectional studies. However, there was no qualitative study that explained people’s perceptions in depth [11].

There is limited research on the impact of preventive measures, including lockdowns and other actions taken during infectious disease outbreaks, in Saudi Arabia, and since the COVID-19 lockdown was unprecedented, there is no research yet about its impact in Saudi Arabia. Through understanding the Saudi community’s perceptions and impact, we can develop tailored interventions that can fulfil two main objectives of our 2030 vision: a vibrant society with a strong foundation in caring for families and empowering our society, as well as an ambitious nation that is responsible for its individual lives and society [12].

Understanding the experiences of different cultures and the application of preventive measures during a pandemic provides critical evidence needed to grasp the global situation and prepare for future health crises. A comprehensive understanding is vital for building resilience against future pandemics, ensuring that global responses are not only scientifically sound but also culturally sensitive and socially inclusive. It can help foster international cooperation, enhance pandemic preparedness, and ultimately save lives by ensuring that responses are both effective and contextually appropriate [13].

This research is unique due to its qualitative approach, which presents an in-depth understanding of why people behave and how they perceive certain phenomena [14]. Exploring people’s experiences during the early stages of the pandemic can inform policies and practices for future pandemics to support people and anticipate their needs. Hence, this study aims to explore the lockdown’s impact on the Saudi Arabian population during the early stages of the pandemic.

## Materials and methods

Study design

This is a qualitative study using semi-structured interviews. Data were collected from April to June 2020. Interviews were analyzed using reflexive thematic analysis [15].

Context

The Saudi Public Health Authority in Saudi Arabia published a guideline in January 2020 on COVID-19 preventive measures [16]. On March 20, 2020, the total number of cases was 344, which led to the closure of the two holy mosques [10]. On April 26, 2020, Saudi Arabia's government declared a partial lockdown, initiating a phased relaxation of preventive measures. This allowed intra-city movement, excluding Mecca, from 9 a.m. to 5 p.m. under specific conditions (such as a ban on social gatherings of more than five individuals). Violations of the curfew carried hefty fines, and repeat violators faced imprisonment. Curfew passes were issued to essential workers and those in need. Additionally, residents were permitted one hour of walking daily within their neighborhood during curfew hours. On May 19, 2020, the cumulative number of deaths was around 100 [10]. However, the reported number of new cases was decreasing in comparison to the recoveries. Hence, on May 31, 2020, the lockdown restrictions were further eased, permitting free movement from 6 a.m. to 8 p.m. across and within regions. This reopening phase enabled people to return to work, reopen mosques, and resume domestic travel. By June 21, 2020, three days after the final interview for this study, the lockdown was fully lifted [10,17].

Study procedures, including participants' recruitment

Researchers sent a survey to participants during lockdown [17]. The survey's aim was to investigate the public's perception of COVID-19 and its preventive measures [17]. A convenience sampling procedure was used to collect respondents. The survey was sent via email and WhatsApp [17]. Participants at the end of the survey were invited to participate in telephone semi-structured interviews to delve into their experiences with the lockdown and other preventive measures during the pandemic. Participants who agreed received an information sheet and consent form. We arranged a convenient time and date for the participant to conduct a telephone interview. Interviews were audio-recorded and transcribed verbatim. The first interview took place on April 28, 2020, and the last interview was conducted on June 18, 2020, when data saturation was reached. Interview times ranged from 15 to 60 minutes. Interviews were conducted in Arabic, and for non-Arabic speakers, it was done in English.

Inclusion and exclusion criteria

Adults who are over the age of 18 and have lived in Saudi Arabia for more than six months or are Saudi citizens were included. Only those who could not read or speak in Arabic or English were excluded.

Instruments description

The topic guide consisted of questions about people’s perceptions regarding the effectiveness and feasibility of preventive measures on a personal and community level, as well as barriers and facilitators to complying with preventive measures. As well as explore its impact on them personally (Appendix 1 for the English and Arabic versions of the topic guide).

Data analysis

To ensure the accuracy of the transcripts, each was checked against the recordings, and any personal information relating to the identity of the participants was removed. The data were transcribed by a professional transcriber. Once interviews were transcribed, five were checked to ensure the accuracy of the transcription. All interviews were managed using the ATLAS.ti web software. Reflexive thematic analysis was used [15]. The first step in the analysis involved familiarization with the data in parallel to data collection, listening to the audio recordings and reading the transcripts a number of times, gaining an overview of the substantive content, and identifying topics and areas of interest while inductively coding the data. This was followed by generating initial themes. The initial themes were reviewed, refined, and developed into final themes and sub-themes. Throughout the process of coding, theme allocation, and writing up, transcripts were revisited to check every stage of the process.

Ethical and safety considerations during conduction of the study

The King Saud University Institutional Review Board (reference number: KSU-HE-20-143) and the King Fahad Medical City Institutional Review Board (reference number: 20-298E) approved the study procedures. An informed consent form and information sheet were sent and discussed with participants prior to the interview. We did not provide any incentives or rewards for participation. Confidentiality and anonymity of the participants’ identities and accounts were protected throughout all stages of data collection, analysis, and result presentation. As the interview questions will explore people’s perceptions, compliance, and behaviour with regard to personal and community-level preventive measures, participants were assured that they would not be judged for expressing any personal views or experiences. We provided participants disclosing any negative experience or showing any signs of distress with appropriate signposting and encouraged them to contact relevant services. Participants were given a choice: to withdraw from the interview or to pause and resume at a later time. The researcher explained and maintained confidentiality between participants throughout the data collection process to facilitate discussion about the topic. The legal limits of confidentiality were clearly explained at the start of the interview (i.e., any potentially identifiable data will be anonymized before publication and dissemination and throughout the process of data analysis and writing up results), while ensuring that participants understood that in exceptional circumstances relating to the safety of the participant or others, confidentiality will have to be broken. However, this was not necessary for this study.

## Results

A total of 28 interviews were conducted with 18 females and 10 males. Four of the participants were non-Saudis. Ages ranged from 18 to 65 years old. One of the participants was infected with COVID-19. Table 1 provides the background of the participants.

**Table 1:** Participants' characteristics

Participant #	Age (years)	Gender	Nationality	COVID-19 +	Education levels	Marital status	Employment status
P1	49	F	Saudi	No	Bachelor	Married	Full-time employed
P2	24	F	Saudi	No	Bachelor	Married	Part-time employed
P3	21	F	Saudi	No	College student	Single	Student
P4	37	M	Saudi	No	PhD	Married	Full-time employed
P5	21	F	Saudi	No	College student	Single	Student
P6	25	F	Saudi	No	Bachelor	Single	Full-time employed
P7	27	F	Saudi	No	Bachelor	Single	Full-time employed
P8	22	F	Saudi	Yes	College student	Single	Student
P9	64	M	Saudi	No	Bachelor	Married	Retired
P10	30	F	Saudi	No	Bachelor	Single	Full-time employed
P11	46	M	Non-Saudi	No	Master	Married	Full-time employed
P12	47	M	Non-Saudi	No	Master	Married	Full-time employed
P13	25	F	Saudi	No	Master student	Single	Student
P14	21	F	Saudi	No	College student	Single	Student
P15	33	F	Non-Saudi	No	Bachelor	Single	Full-time employed
P16	24	M	Saudi	No	Bachelor	Single	Unemployed
P17	23	F	Saudi	No	College student	Single	Student
P18	30	F	Saudi	No	Master	Married	Unemployed
P19	45	F	Saudi	No	PhD	Divorced	Full-time employed
P20	44	M	Saudi	No	Bachelor	Married	Full-time employed
P21	42	M	Saudi	No	PhD candidate	Married	Student
P22	35	M	Non-Saudi	No	PhD	Married	Full-time employed
P23	24	F	Saudi	No	Bachelor	Single	Unemployed
P24	26	F	Saudi	No	Master student	Married	Student
P25	34	F	Saudi	No	Master student	Single	Not specified
P26	40	F	Saudi	No	PhD candidate	Married	Part time
P27	22	M	Saudi	No	College student	Single	Student
P28	22	M	Saudi	No	College student	Single	Student

Two main themes emerged from the data, with three sub-themes each, as shown in Table 2. 

**Table 2:** Summary of themes and subthemes

Main theme	Subthemes
Personal impact	Emotional-based coping (reaction and effect); behavioural changes (proactive and sustained); role responsibility (adaptation and reshaping)
Socio-political impact	Governmental action perception and outlook; healthcare sector's accessibility and burden; societal norms (present, emerging, and future)

Personal Impact

Emotional-Based Coping (Reaction and Effect)

People’s emotional coping ranged from positive/adaptive to negative/maladaptive mechanisms. The negative one dominating the interviews was worry. Worry was mostly about family or loved ones; that participants could infect their loved ones (especially the elderly) with COVID-19 (P23, P13) or about the psychological effect of the lockdown on them specifically for those living alone. The worry about the family was extremely difficult for those whose family were living in another country. Or those who were in the healthcare sector, one of the essential workforces allowed to go to work (P26, P24). Their partners were worried about acquiring the infection from their spouses and infecting their young children.

Worry increased when the participants checked the daily update of case numbers from the beginning of the pandemic and before the lockdown. The daily update was a source of worry for participants, especially if it was local (P13). Participants could explain part of this worry as a concern about the healthcare capacity to accommodate severe cases (P11) or how they perceive the numbers; one participant described the first reported case as more distressing than when it reached a thousand (P4). Another participant was worried about the increase in numbers but relieved at the same time, perceiving that the increased numbers meant better diagnosis and case-tracking procedures (P1). The numbers being reported were also either a cause of concern or a cause of relief. One participant, P12, explained this by starting to focus solely on the rate of recovery as he perceived it as an indication of the health system improving. One participant kept on checking critical cases, describing them as primarily an indication of people’s level of awareness (P26). Another participant felt that worrying about numbers was affecting his health, so he stopped checking them (P11).

For some participants, worry increased due to fear of exposure and the disease; some participants were afraid of the severe complications, including being hospitalized (P4) or death and how that can affect their children or family (P1), and of being carriers and exposing their loved ones (especially parents) to it. People translated that fear into actions by adhering to preventive measures and notifying authorities of those not adhering. People became cautious due to fear since the first case was announced and before the lockdown (P4). One participant started avoiding social gatherings and worked from home (P4). Another participant described how any food delivery is now a conscious decision of whether he could prepare it himself instead of ordering, and that he disinfects orders (P6). That conscious decision-making existed for other preventive measures, such as covering the nose and mouth when sneezing (P8) or going out to buy necessities (P11 and P3). Fear increased or was worse when there was uncertainty about the disease’s spread, medical information, and what it means for the future in terms of regulations and lifestyle.

Another coping mechanism that was experienced by the majority of participants was frustration, and the reasons behind this feeling were different. The majority of participants were frustrated with people, especially family members, who were non-adherent or tried to work around the lockdown procedures. They explained this non-adherence mainly as ignorance; some participants even attributed the government’s decision to enforce lockdown to those non-adherents. Another source for frustration was conflicting and unclear news and research, with the latter frustrating a participant with what he called “academic hype.” He defined it as academic publishing terms and unrealistic figures. Other sources of frustration included the inaccessibility of vulnerable groups (children, those with chronic diseases or disabilities, and those with mental illnesses) to healthcare services, work and study load and responsibilities, difficulty in accessing living essentials such as groceries, inaccessibility to religious sites, unclear guidelines for human resources, travel regulations for those whose family were abroad and travel ticket refunds, and small-space accommodations (i.e., flats vs. villas).

A few participants described feeling angry; their anger was directed towards nonadherent people or towards officials who they perceived were unable - at the start of the pandemic - to organize the situations for those citizens stranded outside the country. One participant also described the ones around them feeling more irritated and angrier than before (P7).

Some participants practiced avoidance by trying to avoid reading or listening to information about COVID-19 or checking the case number updates. One participant just tried to avoid thinking about the social changes that would happen, especially not being able to have family gathered during Eid (Islamic holiday) celebrations. 

Some participants expressed feelings of sadness for not being able to visit others, seeing the empty streets, praying in the holy mosque, and attending funerals to pray for those who passed away.

Some participants experienced feelings of loneliness clearly; those who did not have their family with them or did not have partners, “someone to rely on,” as Participant 19 described, were very lonely. They felt like prisoners in their own houses; they expressed their need for human social or emotional contact. It was, as expressed by participant 463, “very problematic” to be in a lockdown away from social interaction.

As for adaptive mechanisms of coping, a few participants practiced acceptance of the current situation, whether it was the pandemic, lockdown, or the new norm of practicing preventive measures. One participant who was in quarantine for having COVID-19 described how she accepted her situation - even though it was boring as she labelled it - but it was the best option for her not to infect those she loves and to focus on getting better (P8).

Some participants adapted by using cognitive reframing by looking at the positive side of their current situation. Some participants were grateful for the government actions and the healthcare sector personnel efforts they have witnessed. And some felt that they were privileged to be in a national lockdown because it protected them. Indeed, many participants perceived a bright side to their experience, including improved time management for personal and professional development, protection from infection, avoidance of unnecessary social obligations, increased intimate time with the nuclear family, application of preventive measures in retail spaces and homes, increased health awareness, increased reliance on digitalized platforms and options, and increased pride in national identity (patriotism). Some participants emphasized their appreciation of quality time with their family, social cohesion, the essential task force (e.g., nurses, couriers, etc.), leadership, and free time. This experience increased their sense of community and their own sense of responsibility for their actions. Few participants were optimistic as well about the coming days, saying that it would be better despite feeling apprehensive or scared (P14) and that the efforts implemented and people’s adherence indicated success in controlling the disease at the beginning of the pandemic (P28).

Being content was a feeling that counteracted the feelings of anxiety and stress brought on by the pandemic. Hence, a few participants experienced contentment or consciously tried to achieve it. One participant described how content she felt with any task or achievement she made during lockdown (P6). Two other participants described how they felt very peaceful when the lockdown was enforced and that they were not worried about anything, not the healthcare system being overwhelmed or not having enough essentials (P1 and P26).

Behavioral Changes (Proactive and Sustained)

The enforcement of the lockdown at the beginning of the pandemic resulted in changes in the participant's perception of the disease and the implementation of the recommended preventive behaviors. But even before the lockdown, some participants were adherent to preventive practices because of media attention and inherent awareness due to personal attributes such as medical background (P4). Behavioural changes concentrated on adopting preventive measures for those in lockdown, and those who continued to work outside their homes while in lockdown were also practicing preventive measures when they returned, such as wearing masks, social distancing from their family members, etc.

Adhering to preventive measures meant that people also had to change their behaviour and shift towards digital solutions and home-based alternatives for different aspects of their lives, such as socializing digitally, studying and working remotely, practicing home-based fitness activities, and shopping online.

Role Responsibility (Adaptation and Reshaping)

There was an apparent contemplation of roles and identities during this crisis, specifically for parents and caregivers. There were dual challenges: managing their own adaptation and catering to the needs of their dependents. With their roles extending and encompassing many functions that were facilitated by others, such as parents, they had to be educators, full-duty caregivers, and entertainers for long periods of time with limited support; for caregivers of the elderly and the sick, the support decreased, and they had to ensure the safety and well-being of their dependents with limited access to services.

Being the parent of a young child during that period was a particularly difficult experience. Parents struggled with how to entertain their children during lockdown and motivate them to do their tasks and study while adhering to preventive measures. One parent used digital games to motivate them to do their tasks; others were forced to extend their TV time or try to engage them in indoor motor activities. Parents, along with worries about themselves, were worried about the effect of lockdown on the social lives of their children and their mental health, especially for those who were living alone. One parent, whose spouse was a health professional, really struggled with deciding whether to expose herself and her child to her partner, fearing they might get infected.

Socio-political impact 

*Governmental Action Perception and Outlook* 

Lockdown partial approval started when the interviews were conducted, so it gave participants a chance to express their true feelings and perceptions about it. Participants viewed the lift as a necessary element for life to go back to normal, gradually allowing people more time to come and go with fewer limitations and to practice living with the virus. As participant P1037 said, “It is like a new guest that we have to live with,” but the use of the word guest here overlapped with others’ perception that perhaps the government’s easing of lockdown is a cause of celebration that life will get back to pre-COVID-19 normality. 

The lockdown brought a lot of challenges to work and study that the government tried to reduce, but as a new experience, some issues were unexpected or they were unprepared for. For work, there was a perception that if a person is in lockdown, then they are accessible to work all the time. The work-life balance was lost for a lot of people working from home and for students, specifically those who were facing technological challenges because of the pressure placed on the unprepared infrastructure, as educational institutes had to allow different time slots for students for examinations to avoid system crashes. For others, there was no clear guideline on how to proceed with their work, especially those that needed human contact, like private businesses, fieldwork, and so on. If people needed to get clearance from the government, this was also delayed because of the slow administrative processes in certain cases.

Government regulations at the beginning of the pandemic were mostly clear, but the reasons behind the decisions were unclear. As a result, there was confusion and speculation that certain individuals may have been directly impacted financially and personally. Participant P20 said, “For example, I had to close the warehouses because I did not understand if it was allowed or not, and the municipalities were not answering my queries. The municipality itself lacked guidance and clarity. It doesn't know itself; it doesn't have a plan. And this is what caused confusion, forcing me to close."

However, the Ministry of Health’s daily report of cases and advice was a credible source for most participants who checked it regularly, if not daily, at the beginning of the pandemic and used it as the official, trustworthy source of information. Participant P7 said, “The Ministry of Health helped us to the extent that people now know how to act. They provided a number for COVID-19-related queries. If you experience any symptoms, who do you turn to? There's now some awareness about the procedures that need to be followed. It is not just about health awareness with accurate health information but also about the procedures - what we will do, what will happen, and how long it will take. All of this has been helpful, thankfully."

There was a sense of gratitude and trust in the government based on their actions during the early stages of the pandemic. These actions included clear messages of protection and combating, as much as possible, any issue that might affect citizens' and residents' health and finances. Participant P4 said, “Without a doubt, thankfully, I say thanks to God that we are lucky with our country, which has preserved the human being above all else.“

Some participants expressed their hope that certain procedures and actions would extend beyond the pandemic and after the lockdown to counteract or reduce the effects of possible future pandemic health and financial effects. This includes applying preventive measures in crowded places, increasing the health system's capacity, and using technology better, especially in education, business, and commercial areas. A few participants expressed that this experience signifies the need for the government to develop a clear plan in case of future pandemics and to utilize the roles of epidemiology, public health, and health awareness.

Health Care Sector’s Accessibility, Burden, and Role

Healthcare sector accessibility and functioning were a dominant topic for most participants, specifically accessibility during lockdown. Participants who had a chronic disease or a relative/loved one with a chronic disease were really worried about their inaccessibility to their treating physician. Some had no method of contacting their physicians; their prescriptions were difficult to get; private clinics closed, so accessing their physicians there was impossible during the full lockdown; and health professionals whose families were in another country would leave to join them, creating a gap in human resources. Another issue that worried participants was the burden facing the health sector. There was a worry that the health system was not ready, that the focus was on COVID-19 rather than other diseases or health conditions, and that the capacity for receiving patients might not be enough. But it was not the system alone but also the healthcare professionals, their daily experiences, and how it affected their emotional and physical health, as well as their social ties, as they were constantly worried about infecting their loved ones. One participant mentioned that the health sector’s burden may increase not due to an increase in cases only but a decrease in non-Saudi-based health professionals who would want to travel and join their family in another country.

The roles in the health sector included two main functions that participants mentioned: raising awareness and providing treatment and care. The first function was prominent through the daily news conference done by the Ministry of Health’s representative, which was watched almost daily by most participants as they monitored the number and geographical disturbance of cases. They absorbed the preventive measures recommendations that were explained to them directly or through the use of storytelling (i.e., the use of true stories of cases). Raising awareness was tailored to the different social groups and included ethical minorities. And they acted as a credible source for any fake news that would spread in the community (P11, P5). The other function was the care they provided to severe cases in hospitals or quarantined patients; at the beginning of the pandemic, only supportive care and emotional support.

Participant P8 said, "They're doing something unforgettable with remarkable efforts. I'm feeling claustrophobic and emotionally drained, but when I talk to someone who reassures me, it really helps. They deserve thanks for tirelessly responding to people's inquiries despite being exhausted themselves. It makes me feel deeply grateful and proud, wishing I could be part of their efforts."

Societal Norms (Present and Emerging)

Awareness of a person’s responsibility for disease spread was one of the impacts of the lockdown and other governmental procedures/regulations, and officials such as the minister of health and the king’s speech affected them. Awareness also took another form, which is awareness about available services and the rights to access them as citizens or residents. That included, but was not limited to, rights as consumers of goods, delivery and online services, and free healthcare, including free diagnostic tests and hospital treatment. And awareness of people’s role in limiting the pandemic and facilitating the lifting of the lockdown by reporting those who break the rules and consumer good exploiters.

Some participants discussed how awareness by itself was not enough to change behaviour, as there might be barriers to that. A few participants mentioned social pressure as a barrier to specific preventive measures, such as social distance, and how they can be stigmatized as unnecessary or obsessed with cautiousness, while others mentioned that sustained motivation to adhere to the measures was a difficult thing for many.

Respect for the elderly is an integral part of social culture, and physical displays of affection are one aspect of respect that was difficult to implement for many participants. They were worried about how it would be perceived by the elderly. Participants highlighted the effect of gender as they mentioned how being male was linked to outside gatherings and the difficulty of being in lockdown was more for them. 

Awareness was also a cascading consequence of the media coverage of COVID-19 and the strict preventive measures applied by the government. This awareness had an effect on people's hygienic behaviours, where people discussed how they would depend on the use of things inside their home instead of using outside services such as coffee, shaving, manicures and pedicures, personal instruments, and so on. 

The dependence on technology for communicating and accessing services increased during this period and varied, according to many participants. They used it to get health consultations, for education, for delivering goods, and for communicating.

The spread of fake news and studies during this period made a few participants more aware of where and how to access accurate information.

Some participants noted an impact on the social culture. This impact took many facets; it included an observed decrease in physical interactions (e.g., handshakes) and gatherings, respecting boundaries, outcome correspondence for interrelationships (P19), appreciating the nuclear family, empathy towards emotional fragility (P17), social cohesion, appreciating the essentiality of frontline workers that were previously looked down at, increased introversion, and digitalization of communication.

There was cultural stereotyping when a few participants talked about the possibility of the spread of COVID-19 among ethnic groups within the community due to their limited knowledge and understanding of the pandemic and their rights to access free healthcare.

The limited opportunity for social gatherings allowed participants to focus on their religious practices in the comfort of their homes and have more time to connect with their beliefs, especially as lockdown occurred during the holy month of Ramadan for Muslims. These religious beliefs also strengthened the shared identity among many participants as a single community responsible for each other and empathetic towards their shared struggles (P26).

## Discussion

This study provides the first - to the best of our knowledge - lived experience of the Saudi community during the early days of the pandemic and the unique experience of lockdown. The recording of the lived experiences builds on the evidence of the multiple realities lived by different communities and societies across the world and how preventive measures and the social and economic situation of countries play a role in the impacts felt by the country and its residents during difficult times. The study shows that there were personal and socio-political impacts narrated by the participants. The first section primarily focuses on how people experienced emotional regulation in the early days of the pandemic, explaining this phenomenon through the transactional theory of stress developed by Lazarus and Folkman [18]. Emotional coping can happen through adaptive and maladaptive mechanisms, especially in difficult, if not impossible, to change situations that preclude and include perceived threats (e.g., health risks, loss, uncertainty, etc.) [18]. There were a variety of emotional responses and reactions, and they were based on or led to cognitive or behavioural actions. For example, worry, the seemingly dominant emotion among participants, might be the link between appraising the lockdown and pandemic threat and the adaptation of preventive measures, as the original cross-sectional study [17] showed that 74% of participants were worried about COVID-19, but this worry was not mainly due to their fear of the disease (only 16% thought the infection would be severe or life-threatening), but a variety of reasons, as explained in this study, were the basis for the high level of worry. Indeed, understanding the different coping styles and patterns can assist in identifying the differences in the psychological experience of people in such a situation [19]. One study found that positive thinking and social support were positive predictors of well-being [20]. Behavioural changes, another theme identified in this study and seemingly linked to emotional coping, were obvious in the plethora of changes they have undergone to adapt to the changing circumstances. It showed flexibility that depended on digitalized [21] and home replacement solutions. 

For caregivers, their responsibilities for their roles were an important element that presented the dual challenges faced by them in such circumstances. These challenges probably play an important role if they are not the main factor that is attributed to the negative mental health issues arising among families with children and caregivers [22-24].

There were mixed responses to the government's partial lockdown lift, seen as both a step towards normalcy and a source of concern. Government regulations were initially clear but lacked clear rationale, causing confusion. However, the Ministry of Health's consistent updates were a credible source of information, building trust in government actions [25]. Most studies of the knowledge and attitude of the Saudi population towards government regulation during this period showed approval and trust [26,27]. This trust should be empathized when creating a clear communications strategy to allow for a higher level of engagement [28].

The lockdown brought challenges, especially in work-life balance, and technological hurdles in education, especially for families. One review that looked at organizational and occupational health found that work-life balance was affected, especially for workers with either heavier loads and no support from management and/or colleagues or those with family duties [29].

Healthcare accessibility was a significant concern, particularly for those with chronic diseases. The health sector was burdened, focusing mainly on COVID-19 and raising worries about neglecting other conditions. There has been a documented decrease in seeking healthcare services not associated with COVID-19 [30,31]. However, online appointments showed a rapid increase [31]. Indeed, a qualitative study of patients’ experiences with digitalized primary care services in Saudi Arabia during the pandemic showed a positive attitude towards digitalized services [32]. 

Societal norms shifted notably. Awareness about disease spread and service rights increased, though social pressures sometimes impeded preventive measures. The pandemic influenced cultural practices, technology reliance for services, and information discernment. Social behaviours evolved, with reduced physical interactions and greater appreciation for community and frontline workers. Overall, the pandemic's impact was far-reaching, affecting government actions, healthcare, and societal behaviors. Indeed, studies have shown that new norms have a positive association with the risk of a problem; if the risk increases, so do social norms to reduce the threat of COVID-19 [33]. However, social norms tightening and increasing need interventions to speed up the process as there are factors that might influence it [33], and in this study, it seemed that social pressure might have played a role in impeding the culture of adherence to preventive measures.

Recommendation

Understanding the coping mechanisms and strategies for a community can enhance efforts to improve resilience in the face of difficult situations [34] and the gaps that need to be addressed for future crises. Understanding the experiences and impact (specifically the coping style) within a specific cultural context allows for an untangling of the factors associated with experience and mental health [19], which can facilitate the development and evaluation of effective interventions [34]. There is a need to understand the changes that occur in the tightening or loosening of social norms and changes in the risk of disease [33]. Further qualitative research is needed to understand and detail the impact and develop tailored. interventions based on people’s experiences and needs.

Strengths and limitations

This is the first study to our knowledge to explore the experiences of people during the early stages of the pandemic. It provides context and facilitates an understanding of the numerous cross-sectional studies done during that period [11]. But it has limitations, and the main one is the possibility of social desirability bias. However, social desirability can be reduced through the use of different strategies throughout topic guide development and the interview [35]. Recruiting participants who only responded to the online survey might have led to the exclusion of certain groups, such as the elderly, thereby increasing the digital divide.

## Conclusions

The qualitative analysis of experiences during the COVID-19 lockdown in Saudi Arabia illuminates the profound and varied impacts on individuals' emotional well-being, behaviours, and perceptions of socio-political measures. The study highlights the resilience and adaptability of the Saudi population in the face of unprecedented challenges, including significant emotional coping mechanisms and behavioural adjustments. It also underscores the critical role of government communication and policy-making in managing public health crises. The findings suggest that future interventions should be culturally tailored, supporting individuals' emotional and behavioural needs while enhancing the healthcare system's accessibility and resilience. Moreover, fostering a clear and trusting relationship between the government and the public is essential for effective pandemic response and preparedness. This research contributes valuable insights for developing more responsive and inclusive strategies in anticipation of future health emergencies.

## Appendices

English and Arabic Version of the Topic Guide

Date and time:

Location:

Interviewer name:

Participant ID number:

Introduction:

“Thank you very much for agreeing to take part in this interview. My name is… I am a researcher from King Saud University. The aim of this interview is to understand your experience with COVD-19 and how the various preventive measures may impact your daily life. Understanding that can help us better prepare for any future outbreak efficiently and effectively. This interview will take no more than 40 minutes. Please feel free to express your opinion as honestly as you can.

Your views would be extremely valuable.

Before we start, do you have any questions about the consent form you’ve already completed or the study? 

I would like to remind you that at any time you feel uncomfortable, would like to stop, or don’t want to answer certain questions, we can do that. The choice goes back to you.

This conversation will be recorded so that we can concentrate on talking to you and listen back later on for analysis/research purposes.

Do you have any question before we start?

"شكرا جزيلا على موافقتك لاجراء هذه المقابله معك، اسمي .... باحثة من جامعة الملك سعود، الهدف من المقابلة هو التعرف على تجربتكم مع جائحة كورونا و أثر الاجراءآت الوقائية على حياتكم اليومية، فهمنا لهذا يمككنا من التجهيز بطريقة فعالة و اقتصاديا و إنسانيا غير مكلفة، كل ماستخبرني به في هذه المقابلة سيكون قيم جدا و ارائك الشخصية و تجاربك و اقتراحاتك جدا مهمه بالنسبة لنا، المقابلة لن تستلزم اكثر من ٤٠ دقيقة ..."

قبل ان نبدا، هل لديك أي سؤال عن استمارة الموافقة على اجراء البحث؟ حسنا، اود تذكيرك باننا بإمكاننا التوقف في أي وقت تريده كما ان الخيار يعود اليك تماما اذا رغبت بعدم الرد على أسئلة معينه ...

سيتم تسجيل هذه المحادثة حتى نتمكن من التركيز على محادثتنا والاستماع لها  لاحقا لغرض التحليل و البحث.

هل لديك أي سؤال قبل ان نبدأ؟

1.      Personal experience during the COVID-19 outbreak in Saudi Arabia:

When did you first hear about COVID-19 and what did you think of it?

Worried? Or not?

What has your experience been during the COVID-19 outbreak?

In the beginning and now?

Has your view changed?  Or Your feelings toward it? 

How and why

News? Rumors?

Have you or someone you know got COVID-19? What was that like?

Do you have any worries or concerns around COVID 19؟

متى كانت اول مرة سمعت عن فيروس كورونا الجديد ؟ و ماذا  دار في بالك وقتها؟

كنت قلق/ غير قلق؟ لماذا؟

هل بامكانك التحدث عن تجربتك مع جائحة فيروس كورونا؟ 

في البداية؟الان؟

ماذا تغير عليك؟ نظرتك لها؟ مشاعرك تجاهها؟ كيف و لماذا؟

الاخبار؟ الشائعات؟

هل اصبت بفيروس كورونا او احد من معارفك؟ كيف كانت التجربة؟

هل لديك أي مخاوف اتجاه كورونا؟ هل يعتريك القلق بسببه؟ لماذا؟ 

2.      Preventive measures against COVID-19:

What do you think of the advice on hygiene and individual level preventive measures against COVID-19? What are they?

Effective? Why?

Easy/hard to follow? why?

How was your experience with social distancing/self isolation? 

Easy/hard? Why?

Advantages/disadvantages?

·      what about working from home

How did you cope?

How do these measures affect you/ your family?

What do you think of the measures taken by the government?

Effective?/Do you feel the govt has taken the right measures? Why?

How did they make you feel?

What further support would you like?

ماهو رأيك باجراءات او نصائح الوقاية الشخصية من فيروس كورونا؟ و ماهي؟

فعالة؟ لماذا؟

متعبه؟ لماذا؟

سهل/صعب اتباعها؟ لماذا؟

ماهي تجربتك مع الابعاد المجتمع / العزل الصحي؟

سهل/صعب؟ لماذا؟

إيجابيات/سلبيات؟

كيف استطعت التاقلم معها؟

ماذا عن تجربتك بالعمل من البيت؟

 كيف كان تأثير الاجراءآت على حياتك الشخصية/ العائلية؟

ماهو رأيك بالاجراءآت الوقائية المتخذة من قبل الحكومة؟

فعالة؟  هل اتخذوا الاجراءآت الصحيحة؟ لماذا؟

ماهو احساسك تجاهها؟ كيف جعلتك تشعر؟

ماهي أوجه الدعم الإضافي التي بودك الحصول عليها؟

3.      Final message /advice to the health sector/ those with COVID-19?

Do have any final message for the health sector, health professionals or those infected with COVID-19?

Thank you for your time.

ماهي اخر رسالة/ نصيحة تود قولها للممارسين الصحيين ؟ النظام الصحي؟ المصابين بكورونا؟ 

شكرا جزيلا لمشاركتك معنا
